# Quinolinic acid toxicity on oligodendroglial cells: relevance for multiple sclerosis and therapeutic strategies

**DOI:** 10.1186/s12974-014-0204-5

**Published:** 2014-12-13

**Authors:** Gayathri Sundaram, Bruce J Brew, Simon P Jones, Seray Adams, Chai K Lim, Gilles J Guillemin

**Affiliations:** Applied Neurosciences Program, Peter Duncan Neurosciences Research Unit, St Vincent’s Centre for Applied Medical Research, Sydney, Australia; School of Medical Sciences, Faculty of Medicine, University of New South Wales, Sydney, Australia; Department of Neurology, St Vincent’s Hospital, Sydney, Australia; Neurodegenerative diseases Research Group, Australian School of Advanced Medicine, Faculty of Human Sciences, Macquarie University, Sydney, NSW 2109 Australia

**Keywords:** Multiple sclerosis, Oligodendrocyte, Quinolinic acid, Excitotoxicity, Neurodegeneration, Neuroinflammation

## Abstract

The excitotoxin quinolinic acid, a by-product of the kynurenine pathway, is known to be involved in several neurological diseases including multiple sclerosis (MS). Quinolinic acid levels are elevated in experimental autoimmune encephalomyelitis rodents, the widely used animal model of MS. Our group has also found pathophysiological concentrations of quinolinic acid in MS patients. This led us to investigate the effect of quinolinic acid on oligodendrocytes; the main cell type targeted by the autoimmune response in MS. We have examined the kynurenine pathway (KP) profile of two oligodendrocyte cell lines and show that these cells have a limited threshold to catabolize exogenous quinolinic acid. We further propose and demonstrate two strategies to limit quinolinic acid gliotoxicity: 1) by neutralizing quinolinic acid’s effects with anti-quinolinic acid monoclonal antibodies and 2) directly inhibiting quinolinic acid production from activated monocytic cells using specific KP enzyme inhibitors. The outcome of this study provides a new insight into therapeutic strategies for limiting quinolinic acid-induced neurodegeneration, especially in neurological disorders that target oligodendrocytes, such as MS.

**Electronic supplementary material:**

The online version of this article (doi:10.1186/s12974-014-0204-5) contains supplementary material, which is available to authorized users.

## Introduction

Quinolinic acid (QUIN) is a downstream metabolite produced through the kynurenine pathway (KP) of tryptophan metabolism [1,2]. In physiological conditions, QUIN is present in nanomolar concentrations and used as substrate by cells to synthesize the essential co-factor nicotinamide adenine dinucleotide (NAD^+^). However, during neuroinflammation, the KP can be chronically or acutely activated through the induction of one of its initial enzymes, indoleamine 2,3-dioxygenase (IDO-1). QUIN is then produced in excess and can kill brain cells including neurons, astrocytes and oligodendrocytes by at least six different mechanisms [2,3]. QUIN has been implicated in several neurological diseases, including multiple sclerosis (MS) [4,5]. The involvement of the KP in neurological diseases is complex, as it revolves around the metabolic balance between excessive production of neurotoxic metabolites, such as QUIN, and neuroprotective compounds, such as kynurenic acid (KYNA) [6,7].

At the cellular level, QUIN is cytotoxic for most of the brain cell types *in vitro* at micromolar concentrations. Cammer [8,9] showed that exposure to 1 mM of QUIN induces cell death in rat oligodendrocytes [8,9]. Similar toxic effects are also observed in primary human astrocytes and neurons at pathophysiological concentrations of 150 nM [10], and more recently in motor neurons at concentrations of 100 nM [11]. Furthermore, this effect can be abolished by using antagonists of the N-methyl-D-aspartate (NMDA) receptor - such as memantine, MK801 and AP-V - implying excitotoxicity as the main mechanism inducing cell death [10,11]. Current evidence suggests only monocytic lineage cells have the ability to produce QUIN [12,13]. Brain cell types, including neurons, astrocytes, pericytes and endothelial cells are likely to uptake QUIN and catabolize it [14-17].

The function of the KP in oligodendrocytes remains to be investigated, although an earlier study demonstrated that IDO-1 and tryptophan 2,3-dioxygenase (TDO-2) are not expressed in human primary oligodendrocytes [5]. This potentially has strong implications for MS pathology. The lack of these two KP regulatory enzymes in oligodendrocytes is associated with a higher cell susceptibility to allogenic T-cell challenge, since IDO-1 plays a crucial role in immune regulation - particularly in suppressing T cell proliferation [18]. The KP profile has been shown to be altered in both MS patients and in experimental autoimmune encephalitis (EAE) mouse models [19-21]. Rejdak *et al*. [19] reported that the concentration of the neuroprotective KYNA in the cerebrospinal fluid (CSF) of MS patients is decreased during the remission stage. This indicates that the KP stays inclined towards neurodegeneration long after inflammation. We hypothesise that in MS the sustained activation of the KP in the CNS leads to an unbalanced production favoring neurotoxic over neuroprotective intermediates.

This study investigates the profile of the KP in oligodendrocytes and characterizes to what extent KP metabolism changes during immune challenge. We have also identified novel approaches to protect oligodendrocytes against pathophysiological concentrations of QUIN. The outcomes of this study could have significant implications for potential therapeutic strategies in MS and other neurodegenerative diseases that involve KP dysregulation with overproduction of QUIN.

## Material and methods

### Cell cultures

Mouse oligodendroglial cell lines (N19 and N20.1) were kindly provided by Dr. A T Campagnoni (University of California, Los Angeles, CA, USA). These cells were conditionally immortalized oligodendrocytes isolated from normal and shiverer primary mouse brain cultures through the use of the retroviral vector pZIPSVtsA58 encoding immortalizing thermolabile simian virus 40 large T antigen that allows for clonal selection by conferring neomycin (G418) resistance [22]. N19 and N20.1 were chosen for their differences in developmental maturity along the oligodendrocyte lineage. N19 represent oligodendroglial cells with a relatively immature or precursor phenotype, whereas N20.1 represent a more mature phenotype. Oligodendroglial cells were cultured in accordance with the previously published method [22,23]. Briefly, cells were seeded and grown in flask coated with poly-L-lysine to confluence at 34°C, the permissive temperature, in DMEM/F12; Invitrogen, Melbourne, Australia) supplemented with 10% FBS, 100 μg/ml of gentamicin, 100 μg/ml of G418 (Invitrogen, Melbourne, Australia) and then shifted to 39°C, the non-permissive temperature that leads to 'differentiated' state for 72 hours. In all the experiments, during incubation at 39°C, the medium was replaced by fresh DMEM/F12 with 1% FBS and maintained for 7 days before treatments.

The BV2 cell line was kindly donated by Dr. T Kuffner (St Vincent’s Center for Applied Medical Research, Sydney, Australia). The culture method for the BV2 murine microglial cells was adapted from Laurenzi and Arcuri *et al.* [24]. Briefly, BV2 cells were maintained in DMEM supplemented with 10% FBS, Glutamax and antibiotic-anti-mycotic solution. The mouse macrophage cell line RAW264.7 was kindly donated by Prof. Nicholas Hunt (University of Sydney). The RAW264.7 cells were cultured based on the method adapted from Watts and Hunt *et al*. [25].

The study conditions were: (1) unstimulated; (2) IFN-γ stimulated oligodendroglial cell lines (N19 and N20.1); and (3) IFN-γ-treated macrophage cell line, RAW 264.7 as a positive control, since macrophages are known to express all the KP enzymes [14]. The study design included 3 time points: 24, 48 and 72 hours based on previous similar studies showing peak mRNA expression of KP enzymes at 24 hours, whilst production of KP metabolites peaks at 72 hours post-treatment (data not shown) [15,16].

### Characterization of KP enzymes by quantitative RT-PCR

The qRT-PCR protocol used has been previously described [26]. The primer sequences used to detect mouse KP enzymes are summarized in Table 1. Primers were obtained from several sources: designed by Dr. Fabrice Magnino (PCR/qPCR specialist Integrated Sciences Pty Limited), a gift from collaborating researchers, Primer Bank (http://pga.mgh.harvard.edu/primerbank/) or designed by using Primer BLAST (web-based NCBI primer designing tool: http://www.ncbi.nlm.nih.gov/tools/primer-blast/) or Primer3 designing program (http://primer3.sourceforge.net/). RPL13 was used as the endogenous reference gene. Data are presented as fold differences in gene expression normalized to RPL13 and relative to the untreated control. Differences in the relative expression of each gene were analyzed using Student’s *t*-test. Statistical significance was accepted at *P* < 0.05. Reported values are mean ± SE of triplicate samples.

**Table 1:** **Primer sequences for qRT-PCR**

**Gene**	**ID**	**Accession number**	**Primer sequence**	
Indoleamine 2,3-dioxygenase	IDO1	NM_008324	FWD	TGT GAA TGG TCT GGT CTC
			REV	CTG TGC CCT GAT AGA AGT
Tryptophan 2,3-dioxygenase	TDO2	BC018390.1	FWD	TGC TCA AGG TGA TAG CTC GGA
			REV	AGG AGC TTG AAG ATG ACC ACC A
Kynurenine aminotransferase 1	KAT1	NM_172404	FWD	GCT TTT CAG CAG GCT ACC AC
			REV	CCA CTG TCA CCA GCA CAT TC
Kynurenine aminotransferase 2	KAT2	AF072376	FWD	GAA CTT CTG TCC TGG CTA A
			REV	CTT GAT TGG GTG GGT AGT
Kynureninase	KYNU	NM_027552	FWD	GAG CAG AGG AGC GTG GCT GC
			REV	GAA CAG GGG CCA CGC GGA TG
3-hydroxyanthranilate 3,4-dioxygenase	3HAO	NM_025325	FWD	TTC AGC CTC ATT GCA TCT
			REV	GAC AGT GTA GGG CTA TGG
Kynurenine 3-monoxygenase	KMO	NM_133809.1	FWD	GGT CGC CTT CAC CAG AAT AA
			REV	ATC CAG GCA GGT CTT CTC AA
Aminocarboxymuconate semialdehyde decarboxylase	ACMSD	NM_001033041	FWD	GAA TAA ATG CTG ACC CAA CA
			REV	TTC ATC CAT CCT TCC AGA C
Quinolinate phosphoribosyl transferase	QPRT	NM_133686.1	FWD	CTG CTC CAA GTC ACC ATG
			REV	CAG AAC CCC AGG AGA TTT
Ribosomal protein L13	RPL13	NM_016738.5	FWD	GAG GTC GGG TGG AAG TAC CA
			REV	TGC ATC TTG GCC TTT TCC TT

### Immunocytochemistry

To characterize the KP in both N19 and N20.1 cell lines, cells were cultured onto slide flasks (Nunc, Rochester, NY, USA) at 34°C and when 70% confluence was reached they were incubated at 39°C for 72 hours. After 3 days, the cells were either left untreated or treated with 100 IU/ml of IFN-γ for 24 hours. The cells were then stained for indoleamine 2,3-dioxygenase 1 (IDO-1) (Biolegend, San Diego, CA, USA), tryptophan 2,3-dioxygenase (TDO-2; generous gift from Dr. CL Miller John Hopkins University, Baltimore, MD, USA), kynurenine 3-monooxygenase (Abcam, Cambridge, UK), QUIN (Millipore, Bedford, MA, USA), picolinic acid (PIC; Abcam, Cambridge, UK), 2',3'-cyclic nucleotide 3'-phosphodiesterase (CNP; Millipore, Bedford, MA, USA) and myelin basic protein (MBP; Abcam, Cambridge, UK) according to previously described protocol [15,16]. The antibodies are summarized in Tables 2 and 3. Untreated cells were stained and used as experimental control for comparison. Experiments were performed in triplicate and independently validated with three biological replicates.

**Table 2:** **Summary of primary antibodies**

**Antibody**	**Dilution**	**Isotype**	**Company**
Myelin basic protein (MBP)	1:100	Monoclonal - IgG	Abcam
2',3'-cyclic nucleotide 3'-phosphodiesterase (CNP)	1:50	Monoclonal - IgG	Millipore
Vimentin (VIM)	1:100	Monoclonal - IgG	Millipore
Glial fibrillary acidic protein (GFAP)	1:750	Monoclonal - IgG	Dako
Indoleamine 2,3-dioxygenase 1 (IDO1)	1:100	Monoclonal - IgG	Biolegend
Tryptophan 2,3-dioxygenase (TDO)	1:100	Polyclonal	[27]
Kynurenine 3-monooxygenase (KMO)	1:100	Polyclonal	Abcam
Quinolinic acid (QUIN)	1:100	Monoclonal - IgG	Millipore
Picolinic acid (PIC)	1:100	Monoclonal - IgG	Abcam

**Table 3:** **Summary of secondary antibodies**

**Antigen**	**Dilution**	**Isotype**	**Company**
Alexa Fluor 488 (green)	1:250	Goat anti-mouse IgG	Invitrogen
Alexa Fluor 488 (green)	1:250	Goat anti-rabbit IgG	Invitrogen
Alexa Fluor 594 (red)	1:250	Goat anti-mouse IgG	Invitrogen
Alexa Fluor 594 (red)	1:250	Goat anti-rabbit IgG	Invitrogen

### Quantification of KP metabolites

Tryptophan, kynurenine, and kynurenic acid were measured using high performance liquid chromatography (HPLC) while picolinic acid and QUIN were concurrently measured using gas chromatography/mass spectrometry (GC/MS) as previously described [11]. Results of tryptophan and kynurenine were presented as kynurenine/tryptophan (K/T) ratio, a reliable measure of KP activation. Production or degradation of the KP metabolites was calculated by subtracting the values of post- and pre-treatment concentrations of the KP metabolites present in the culture medium.

### QUIN toxicity and cell protection assays

QUIN toxicity and cell protection assays against QUIN were determined by calculating the percentage of cell survival rate in comparison to untreated controls using CytoTox 96® Non-Radioactive cytotoxicity assay kit (Promega, Madison, WI, USA). Experiments were divided into four parts:

A) QUIN toxicity on oligodendrocytes: to assess the QUIN toxicity, oligodendroglial cells were challenged with varying concentrations of exogenous QUIN up to 4 μM over 3 time points (24, 48 and 72 hours) and QUIN toxicity assessed. These data were used to determine the LD_50_ of QUIN toxicity and select the appropriate treatment period in subsequent QUIN antagonism experiments (*Parts C and D*).
B) QUIN uptake assays: to further demonstrate the uptake of QUIN in oligodendrocytes, cells were grown in slide flasks (Nunc, Rochester, NY, USA) and treated with LD_50_ dose described in *Part A* for 0, 30, 60 and 90 minutes using protocol adapted from [28]. QUIN uptake was then visualized using immunocytochemistry as described previously [11,28].
C) Neutralization of QUIN with an anti-QUIN monoclonal antibody (mAb): to fully assess the potential of neutralizing QUIN toxicity with an anti-QUIN mAb, we subjected the oligodendroglial cells to 2 different conditions: 1. treated directly on oligodendroglial cell lines with exogenous QUIN followed by varying concentrations of QUIN-mAb with the following three conditions: (a) pre-treatment with QUIN (QUIN-PRE) for 72 hours at LD_50_ concentration followed by the QUIN-mAb for 30 minutes; (b) pre-treatment with anti-QUIN mAb for 30 minutes followed by QUIN (QUIN-POST) at LD_50_ concentration for 72 hours and; (c) concomitant treatment with QUIN and the anti-QUIN mAb (QUIN + QUIN mAb) together for 72 hours. 2. treated with IFN-γ-treated BV2 cells supernatant (endogenous QUIN) on oligodendroglial cell lines followed by varying concentrations of QUIN mAb. Cell death was then determined by measuring lactate dehydrogenase (LDH) in the culture supernatant.
D) Inhibition of QUIN production with IDO-1 inhibitors: to imitate QUIN production during inflammation and immune activation, BV2 cells were stimulated with IFN-γ for 24 hours to induce pathophysiological concentrations of QUIN production. Oligodendrocyte cell line cultures were then exposed to this QUIN-containing BV2 culture supernatant for 72 hours and assessed for QUIN toxicity. Further, the QUIN-producing BV2 cells were challenged with 4 specific IDO-1 inhibitors namely, 1-methyl-D-tryptophan (D-1MT), 1-methyl-L-tryptophan (L-1MT), 1-methyl-D-tryptophan (DL-1MT) and berberine (5,6-dihydro-9,10-dimethoxybenzo[g]-1,3-benzodioxolo[5,6-a]quinolizinium) for 30 minutes to block QUIN production as a potential therapeutic strategy to alleviate QUIN toxicity during neuroinflammation.

### Statistical analysis

Results are expressed as mean ± SE. Differences between treatment groups for RT-PCR, GC/MS and HPLC data were analyzed using Student’s *t*-test. QUIN toxicity study results were compared using linear and multiple regression analysis. QUIN mAb treatment results were analyzed using the Mann-Whitney test. In all experiments a *P*-value < 0.05 was considered significant.

## Results

### KP profiling in oligodendroglial cell lines N19 and N20.1

**Figure 1:**
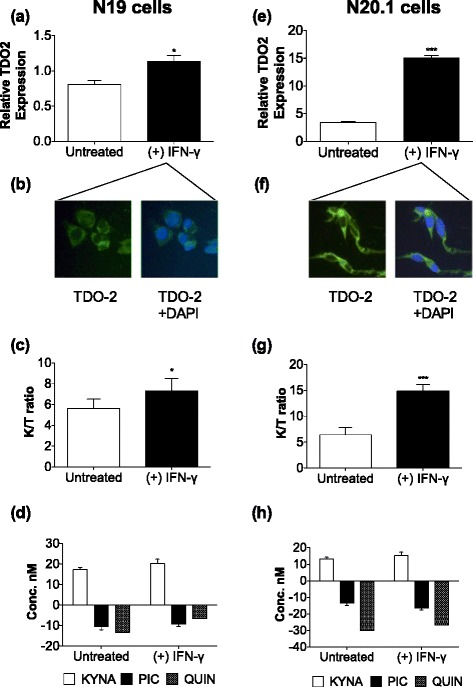
**Characterization of the kynurenine pathway (KP) in N19 (a-e) and N20.1 (e-h) oligodendroglial cell lines.** Levels of TDO-2 transcripts in **(a)** N19 and **(e)** N20.1 cells in differentiated state, with or without IFN-γ treatment. N19 cells **(b)** and N20.1 cells **(f)** showed positive TDO-2 cytoplasmic staining after 24 hours IFN-γ treatment. KP metabolic profile of oligodendroglial cell lines K/T ratio in N19 **(c)** and N20.1 **(g)** and kynurenic acid (KYNA), picolinic acid (PIC) and quinolinic acid (QUIN) profiles of N19 **(d)** and N20.1 **(h)** treated with or without IFN-γ. TDO-2, tryptophan 2,3-dioxygenase; K/T ratio, kynurenine and tryptophan ratio as a measure of IDO-1/TDO-2 activity; KYNA, kynurenic acid; PIC, picolinic acid; QUIN, quinolinic acid. Values are expressed as mean ± SE. ***P < 0.001, **P < 0.01 and *P < 0.05. Student's *t*-test.

We found that both mice oligodendrocytic cell lines N19 and N20.1 have a functional KP. This is evident at gene, protein and metabolite level in N19 (Figure 1a-d) and N20.1 (Figure 1e-h). However, the profile of KP expression in N19 was significantly different from N20.1 (see Additional files 1 and 2). By comparing the levels of mRNA expression for the KP enzymes, after normalization with the endogenous reference gene RPL13, we showed that N20.1 had a much higher level of expression for TDO-2 (*P* < 0.001) than N19 (Figure 1a and e). This result was validated at protein level using immunocytochemistry (Figure 1b and f). Both cell lines showed increased mRNA expression for TDO2 when treated with pro-inflammatory cytokine IFN-γ. In N20.1 cells most KP enzymes were up-regulated by IFN-γ, except ACMSD (see Additional file 1). As expected, treatment with IFN-γ led to an increased KP activation shown by the increased K/T ratio in both cell lines (Figure 1c and g). Interestingly this increase was due to an up-regulation of TDO-2 rather than IDO-1 gene expression in N19.

KAT-1 and KAT-2 expression in both N19 and N20.1 suggests that oligodendrocytes are capable of producing the neuroprotective metabolite KYNA. Indeed, KYNA was detected in the culture media of both oligodendrocytic cell lines following background subtraction (serum supplemented media). Although there was a significant increase in KAT-1 and KAT-2 gene expression in N20.1 cells (see Additional file 1) following IFN-γ treatment, there was no significant difference in the levels of KYNA produced (Figure 1d and h). ACMSD activity was low in both cell lines (see Additional file 1) and no production of PIC was detected in culture media of the cell lines (Figure 1d and h). Moreover, a negative value for PIC (N19: untreated: −10.345 nM, IFN-γ- treated: −9.234 nM; N20.1: untreated: −13.345 nM, IFN-γ-treated: −16.356 nM) indicate post-culture concentrations were lower than that in pre-culture conditions. This suggests exogenous PIC, present in serum supplemented media, is taken up and/or catabolized by the oligodendrocytes. Low QUIN concentrations were also observed in the culture media of both cell types (Figure 1d and h). Along with the elevated activity of QPRT seen (see Additional file 1) this suggests an increased uptake and degradation of QUIN by oligodendroglial cells. To address this possibility we next investigated QUIN uptake by N19 and N20.1 cells.

### Evidence of exogenous QUIN uptake in oligodendrocytes

**Figure 2:**
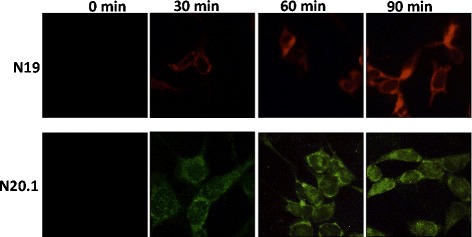
**Quinolinic acid (QUIN) time-course study.** N19 (top row) and N20.1 cells (bottom row) treated with specified concentration of QUIN for 0, 30, 60 and 90 minutes. Treated cells were analyzed by immunohistochemistry for QUIN uptake.

Using immunocytochemistry, we have demonstrated the capability of both oligodendrocyte cell lines to take up exogenous QUIN (Figure 2)*.* This indicates QUIN is catabolized intracellularly in a time-dependent manner as fluorescence intensity was directly proportional to the uptake of QUIN.

**Figure 3:**
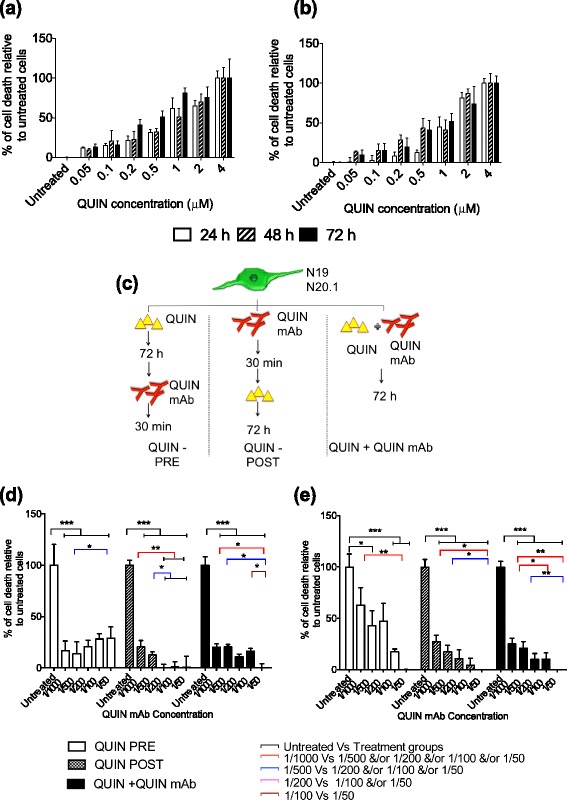
**Neutralization of exogenous quinolinic acid (QUIN) with an anti-QUIN monoclonal antibody (mAb).** The QUIN toxicity assay was investigated in **(a)** N19 and **(b)** N20.1 cells treated with various concentration of QUIN (0, 0.05, 0.1, 0.2, 0.5, 1, 2 and 4 μM) for 24 hours, 48 hours and 72 hours. Lactate dehydrogenase (LDH) activity in culture supernatant was determined. The results were analyzed using linear regression analysis. **(c)** The schematic diagram showed the treatment conditions of QUIN and QUIN mAb on oligodendroglial cells. The effect of QUIN mAb on QUIN toxicity was examined in **(d)** N19 and **(e)** N20.1 cells. The levels of LDH released by oligodendroglial cell lines after 72 hours of incubation with 0.5 μM (N19) and 1 μM (N20.1) of QUIN and varying concentrations of QUIN mAb (30 minutes). The study was performed in triplicate and the error bars indicate SE. ***P < 0.001.

We then further assessed the cytotoxic threshold of such QUIN uptake on oligodendroglial cells. A standard curve, using commercial exogenous QUIN (Sigma-Aldrich, St Louis, MO, USA), was produced to assess the level of gliotoxicity by QUIN on N19 and N20.1 cells. This demonstrated a relatively similar level of tolerance in the two cell lines to QUIN (Figure 3a and b). There was a notable trend indicating dose-dependent QUIN toxicity on oligodendrocytes where the LD_50_ for N19 is 0.5 μM and for N20.1 is 1 μM for 72 hours. These LD_50_ values were chosen as the reference criteria for subsequent QUIN antagonism studies.

### Antagonism of QUIN toxicity with neutralizing anti-QUIN monoclonal antibody (mAb)

Based on the 3 conditions illustrated in Figure 3C and the presence of LD_50_ concentrations of QUIN for 72 hours (determined in Figure 3a and b), linear regression analysis showed that QUIN toxicity on N19 and N20.1 cell cultures was neutralized by QUIN mAb treatments (Figure 3d and e). We found that treatment with anti-QUIN mAb diluted at 1/50 (14 ng/μL) led to complete protection against QUIN excitotoxicity. The pre-treatment with anti-QUIN mAb group was the least effective of the assessed treatments. Comparing the QUIN-PRE treatment groups between both cell lines, the effect of QUIN-induced cell death at LD_50_ in N20.1 was more reversible than N19. A validation study was performed to determine the concentration of anti-QUIN mAb used in the study was not toxic to the cells (data not shown).

**Figure 4:**
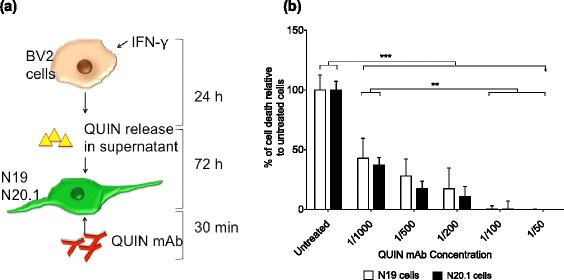
**Neutralization of endogenous quinolinic acid (QUIN) with an anti-QUIN monoclonal antibody (mAb). (a)** The schematic diagram represents the effect of QUIN mAb on IFN-γ-treated BV2 supernatant in N19 and N20.1 oligodendroglial cell lines. **(b)** represents the levels of lactate dehydrogenase (LDH) released by oligodendroglial cell lines N19 and N20.1, respectively after 72 hours incubation with IFN-γ-treated BV2 and varying concentrations of QUIN mAb. The study was performed in triplicate and the error bars indicate SE. ***P < 0.001.

In addition, we also demonstrated that this strategy worked on endogenous QUIN present in supernatants of IFN-γ-treated BV2 microglial cells containing 0.99 ± 0.1 μM of QUIN (data not shown). The glioprotection observed with the QUIN mAb treatment was found to be statistically different (*P* < 0.001) in all the treatment groups when compared to control cells for both the cell types (Figure 4). Among the treatment groups for both cell lines, anti-QUIN mAb at 1/100 (7 ng/μL) or higher concentration showed full protective effect from QUIN-induced cell death.

### Inhibition of immune-induced QUIN toxicity with IDO inhibitors

**Figure 5:**
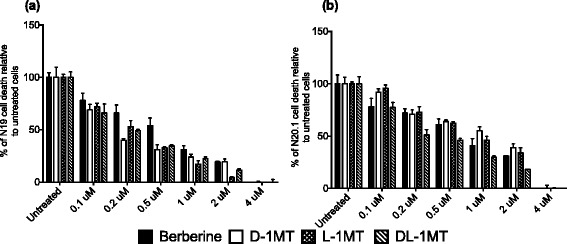
**Effect of IDO inhibition on immune induced QUIN toxicity.** The schematic bar diagram represents the effect of berberine, D-1MT, L-1MT and DL-1MT (0 to 4 μM) on IFN-γ-treated BV2 supernatant in N19 **(a)** and N20.1 **(b)** oligodendroglial cell lines. The levels of lactate dehydrogenase (LDH) released by oligodendroglial cell lines after 72 hours incubation with IFN-γ-treated BV2 supernatant. The study was performed in triplicate and the error bars indicate SE. ***P < 0.001.

We tested 4 IDO-1 inhibitors including the natural compound berberine and 1-methyl-tryptophan (1MT) isoforms D, L and DL for their ability to block IDO-1 and so stop QUIN production by BV2 cells. We found that all four inhibitors were able to decrease QUIN induced toxicity in a dose dependent manner (Figure 5). Prior to study, IDO-1 inhibitors were tested for toxicity concentrations on BV2 cells and were well tolerated above 4 μM (data not shown). At 1 μM, the 4 IDO-1 inhibitors were able to bring the level of cytotoxicity under the LD_50_. At 4 μM, all the IDO-1 inhibitors were able to completely abolish the QUIN-induced cell death in both cell lines. Comparing the percentage of cell death between the 2 cell lines, again we observed that N20.1 cells were slightly more sensitive to QUIN toxicity. A concentration of 0.5 μM was needed to rescue N19 cells to LD_50_ whereas twice as much inhibitor was required to observe a similar effect in N20.1 cells (*P* < 0.001). Berberine was the less effective compound in protecting N19 cells whilst DL-1MT was the most effective in protecting the N20.1 cells during QUIN excitotoxicity.

## Discussion

The KP profile has been extensively characterized in various human cell types including neurons, astrocytes, microglia, endothelial cells and several other immune cells [14-17]. However, the literature regarding the KP in oligodendrocytes is very limited [5]. More importantly the biological function of the KP in oligodendrocytes remains to be delineated. In this study, we showed that murine oligodendrocytic cell lines express all the KP enzymes tested and have a fully functional KP unlike astrocytes, which lack the mid-stream enzyme KMO that converts kynurenine to 3-hydroxykynurenine [15]. However, these data conflict with our previous findings showing that human primary oligodendrocytes lack expression of both IDO-1 and TDO-2 [5]. These discrepancies are likely explained by the fact that KP expression: 1) varies between different species of the same cell type [29-31] and; 2) is significantly different between primary cells and their respective cell lines [5]. It is also possible that the conditional transfection of the oligodendrocytes with simian virus 40 T antigens may alter the KP expression [17]. Furthermore, in this study we showed that KP metabolism is different between two cell lines of the same cell type arising from the same species. N20.1 cells display a more active KP mRNA enzyme expression when compared to N19 (Figure 1). The degree of gene up-regulation by IFN-γ was also different: most of the KP enzymes were induced by IFN-γ in N20.1 cells but only limited to TDO-2, KAT-2 and KYNU in N19 cells. The increase of K/T ratio as a result of TDO-2 up-regulation in these 2 mice oligodendrocytic cell lines was also reported by our group in the human oligodendrocytic cell line, MO3.13 [5]. It was also demonstrated in a recent study that TDO-2 is highly up-regulated in immortalized glial cell lines [32].

The expression of KATs and secretion of KYNA by both cell lines suggests that the oligodendrocytes are likely to have neuroprotective functions, especially during neuroinflammatory events. This is reflected by higher expression of KAT-II in the presence of IFN-γ (see Additional file 1). This concurs with current literature pertaining to the mice model where KAT-2 is demonstrated to be the main enzyme associated with KYNA production in the brain [33]. Expression of QPRT corresponding with catabolic rather than anabolic activity of QUIN further supports the neuroprotective role of oligodendrocytes. In addition, this suggests that oligodendrocytes are capable of taking up exogenous QUIN present in the extracellular matrix. They could, therefore, be involved in the detoxification of pathophysiological concentrations of exogenous QUIN, secreted by activated immune cells. Using immunocytostaining, we further showed a rapid uptake of QUIN by oligodendrocytes occurring within 30 minutes then gradually increasing with time (Figure 2). These results are in agreement with the KP metabolic profile found in human primary oligodendrocytes showing an uptake of exogenous QUIN from media and production of neuroprotective KYNA and PIC [5]. We previously showed that QUIN is cytotoxic to both primary human neurons and astrocytes at concentrations as low as 150 nM and 350 nM respectively [10].

As described above, N20.1 cells display a more active KP compared to N19 cells. This suggests that N20.1 cells are likely to have a higher tolerance to QUIN toxicity, that is a higher capacity to catabolize QUIN. Indeed, this was reflected by the LD_50_ concentration to QUIN toxicity in N20.1 cells with a difference 1-fold higher compared to N19 (Figure 3A). Surprisingly, this was not reflected in the PRE-QUIN treatment group where cells are pre-exposed to QUIN followed by treatment with anti-QUIN mAb (Figure 3D and E). We expected N20.1 cells to have a higher capacity to catabolize QUIN. However, the cell death assay revealed otherwise. It is important to note that the QUIN concentrations based on LD_50_ used in both cell lines are different. It may imply that QUIN at approximately 1 μM concentration range may exert a more permanent and lasting excitotoxic effect. This is regardless of the cells capacity to take up and catabolize QUIN. An important aspect to further dissect is the short-term and long-term effects of QUIN toxicity on oligodendrocytes. This may have important implications for neurodegeneration and repair in situations where the KP is activated for sustained and prolonged periods of time, such as progressive MS. We have previously shown that low micro molar concentrations of QUIN could exert excitotoxicity chronically in human neurons over a period of 5 weeks [34]. Here, our data support that from a previous study by Cammer *et al.* [8] showing acute QUIN toxicity in primary rat oligodendrocytes at micro molar concentrations.

It is interesting to speculate that differences in susceptibility to QUIN-mediated toxicity observed between the two cell lines may also reflect their different maturation states and the associated expression of NMDA receptors. The relatively immature N19 cells, with a lower expression of NMDA receptors, experience reduced excitotoxicity in response to QUIN. Furthermore, it is also possible that there are differences in expression of QUIN transporter molecules and, therefore, varying levels of QUIN uptake possible between cell lines. The characterization of NMDA receptors and the capacity of the QUIN uptake transporter certainly warrant further investigation in oligodendrocyte development.

It is apparent that therapeutic strategies targeting QUIN could prevent excitotoxicity and ultimately significantly attenuate neurodegeneration. In this study, we demonstrated that neutralizing QUIN toxicity using an anti-QUIN mAb could prevent oligodendrocyte cell death *in vitro*. There is still a limited number of studies targeting QUIN toxicity with a neutralizing antibody, despite extensive evidence showing QUIN as a key excitotoxin involved in several neuropathological diseases. This study is the first to propose the use of a mAb to neutralize *in vitro* QUIN toxicity. Based on our findings, the application of neutralizing QUIN with antibodies could be further developed into monoclonal antibody therapy for neurodegenerative diseases such as MS or amyotrophic lateral sclerosis [35]. Monoclonal antibody therapy has gained popularity over the years including the recently FDA approved natalizumab used in MS treatment [36,37].

As described above, chronic inflammatory responses can easily trigger cumulative production of pathophysiological concentrations of QUIN by activated monocytic cells such as infiltrating macrophages and microglia. This likely creates an environment in which neuronal cells are highly susceptible to excitotoxicity. Thus, we investigated further how manipulation of the KP during inflammation could affect the survival of oligodendrocytes towards QUIN-induced toxicity. We attempted to limit the production of QUIN from the BV2 microglial cell line by inhibiting IDO-1, since this enzyme is highly inducible by IFN-γ and will be of pathophysiological relevance during inflammation. Our data show that the use of IDO-1 inhibitors (1-MT and berberine) were able to significantly decrease QUIN production by BV-2 cells and subsequently lead to complete abolishment of oligodendrocyte cell death at a concentration of 4 μM (Figure 5). These results represent another relevant therapeutic strategy for MS using KP inhibitors. An earlier study using Ro 61-8048, a KMO inhibitor, on EAE rats showed a significant alleviation of disease progression [21]. In this same study the authors demonstrated that the advantage of KMO inhibition, in comparison with IDO-1, is that they could obtain both a decrease of QUIN synthesis and an increased production of the neuroprotective KYNA in the brain and spinal cord [21].

The use of IDO-1 inhibitors as a treatment for EAE and MS requires further investigation. The positive outcomes of this study are based on IDO-1 inhibition specifically in activated microglial cells. However, there is no IDO-1 inhibitor able to selectively target cell types. For example, inhibiting IDO-1 in astrocytes, the main producers of KYNA [15] would likely be deleterious. Furthermore, some studies have demonstrated that systemic inhibition of IDO-1 in EAE exacerbates the disease [38,39]. It is likely that the timing of intervention is important in this context: normal KP activation certainly suppresses aberrant immune responses and inhibiting this effect is unwanted in autoimmune disease. However, excessive KP activation produces neurotoxic metabolites, so preventing this dysregulated activation of the KP is clearly neuroprotective. Some KP metabolites have critical roles in the regulation of the T-cell activity responsible for autoimmune disorders [39-41]. This further supports the notion that the homeostatic activation of IDO-1 in EAE does have beneficial outcomes. However, its continued inhibition could also have significant negative effects in progressive disease.

It is technically challenging to deliver IDO-1 inhibitors specifically to activated monocytic cells. However, cell-specific targeting might be possible using nanoparticles [42]. Another possibility will be to deliver multiple targets of KP modulators that can limit QUIN production whilst increasing KYNA and PIC. Platten *et al*. [43] demonstrated that tranilast (Rizaben), a synthetic analog of the KP intermediate 3-HAA, was able to fully abolish EAE by skewing the immunological profile from pro-inflammatory to immunosuppressive [43]. Combination therapy targeting KP enzymes and/or metabolites appears to be a promising therapeutic for EAE and MS but requires further investigation.

## Conclusions

In conclusion, our data show that the oligodendrocytes express the major components of KP and, importantly, are capable of catabolizing exogenous QUIN. Therefore, they are likely to play a role in the detoxification of excessive QUIN associated with a chronic immune-activated KP in monocytic cells. More importantly, QUIN is toxic to oligodendrocytes at pathophysiological concentrations. Herein, we demonstrated two potential therapeutic strategies to overcome the toxicity of QUIN by directly neutralizing with anti-QUIN mAb and indirectly with KP inhibitors to limit QUIN production. This work presents novel therapeutic insights into excitotoxicity and neuroprotection for oligodendrocytes and other brain cell types.

### Main points

- First characterization of the kynurenine pathway (KP) in oligodendrocytes
- Oligodendrocytes take up QUIN
- QUIN is toxic for oligodendrocytes
- Exogenous QUIN toxicity can be neutralized by using a monoclonal antibody against QUIN and specific KP inhibitors leading to increased oligodendrocyte survival

## Additional files

Additional file 1
**Expression of kynurenine pathway genes in oligodendroglial cells.** Levels of transcripts in (a) N19 and (b) N20.1 oligodendroglial cell line in differentiated state, with or without IFN-γ treatment. Dots represent mean value from four different experiments. The levels of transcripts from the genes encoding are IDO1 (indoleamine 2,3-dioxygenase), TDO2 (tryptophan 2,3-dioxygenase), KAT1 (kynurenine aminotransferase1), KAT2 (kynurenine aminotransferase2), KYNU (kynureninase), ACMSD (aminocarboxymuconate semialdehyde decarboxylase) and QPRT (quinolinate phosphoribosyl transferase). Horizontal line indicates median values. ****P* < 0.001, ***P* < 0.01 and **P* < 0.05. Additional file 2
**Expression of kynurenine pathway protein in oligodendroglial cells.** Characterization of the kynurenine pathway in (a) N19 and (b) N20.1 cells by immunohistochemistry. Cells showed positive cytoplasmic staining of TDO2 (N19 and N20.1) and KMO (N20.1 only) after 24 hours IFN-γ treatment. IDO1, QUIN and PIC were absent in this cells. Nuclei stained with DAPI marker. Scale bar 20 μm. RAW 264.7 cells were used as control for positive-staining of IDO1, TDO2, KMO, QUIN and PIC.

## Abbreviations

KP: kynurenine pathway
MS: multiple sclerosis
KYN: kynurenine
TRP: tryptophan
KYNA: kynurenic acid
PIC: picolinic acid
QUIN: quinolinic acid
mAb: monoclonal antibody
IDO-1: indoleamine 2,3-dioxygenase
TDO-2: tryptophan 2,3-dioxygenase
1MT: 1-methyl-tryptophan
