# Supplementary material for: Quinolinic acid toxicity on oligodendroglial cells: relevance for multiple sclerosis and therapeutic strategies
Source: J Neuroinflammation. 2014 Dec 13;11:204. doi: 10.1186/s12974-014-0204-5 (PMC4302518; doi:10.1186/s12974-014-0204-5)
Supplement: Additional file 1 — Expression of kynurenine pathway genes in oligodendroglial cells. Levels of transcripts in (a) N19 and (b) N20.1 oligodendroglial cell line in differentiated state, with or without IFN-γ treatment. Dots represent mean value from four different experiments. The levels of transcripts from the genes encoding are IDO1 (indoleamine 2,3-dioxygenase), TDO2 (tryptophan 2,3-dioxygenase), KAT1 (kynurenine aminotransferase1), KAT2 (kynurenine aminotransferase2), KYNU (kynureninase), ACMSD (aminocarboxymuconate semialdehyde decarboxylase) and QPRT (quinolinate phosphoribosyl transferase). Horizontal line indicates median values. ***P < 0.001, **P < 0.01 and *P < 0.05. [file 12974_2014_204_MOESM1_ESM.docx]

**Supplementary 1** Levels of transcripts in (a) N19 and (b) N20.1 oligodendroglial cell line in differentiated state, with or without IFN-γ treatment. Dots represent mean value from 4 different experiments. The levels of transcripts from the genes encoding are IDO1 (indoleamine 2,3-dioxygenase), TDO2 (tryptophan 2,3-dioxygenase), KAT1 (kynurenine aminotransferase1), KAT2 (kynurenine aminotransferase2), KYNU (kynureninase), ACMSD (aminocarboxymuconate semialdehyde decarboxylase) and QPRT (quinolinate phosphoribosyl transferase). Horizontal line indicates median values. *** P<0.001, ** P<0.01 and *P<0.05.
