# Supplementary material for: Quinolinic acid toxicity on oligodendroglial cells: relevance for multiple sclerosis and therapeutic strategies
Source: J Neuroinflammation. 2014 Dec 13;11:204. doi: 10.1186/s12974-014-0204-5 (PMC4302518; doi:10.1186/s12974-014-0204-5)
Supplement: Additional file 2 — Expression of kynurenine pathway protein in oligodendroglial cells. Characterization of the kynurenine pathway in (a) N19 and (b) N20.1 cells by immunohistochemistry. Cells showed positive cytoplasmic staining of TDO2 (N19 and N20.1) and KMO (N20.1 only) after 24 hours IFN-γ treatment. IDO1, QUIN and PIC were absent in this cells. Nuclei stained with DAPI marker. Scale bar 20 μm. RAW 264.7 cells were used as control for positive-staining of IDO1, TDO2, KMO, QUIN and PIC. [file 12974_2014_204_MOESM2_ESM.docx]

**Supplementary 2** Characterization of the kynurenine pathway in (**a**) N19 and (**b**) N20.1 cells by immunohistochemistry. Cells showed positive cytoplasmic staining of TDO2 (N19 and N20.1) and KMO (N20.1 only) after 24 h IFN-γ treatment. IDO1, QUIN and PIC were absent in this cells. Nuclei stained with DAPI marker. Scale bar 20 μm. RAW 264.7 cells were used as control for positive-staining of IDO1, TDO2, KMO, QUIN and PIC.


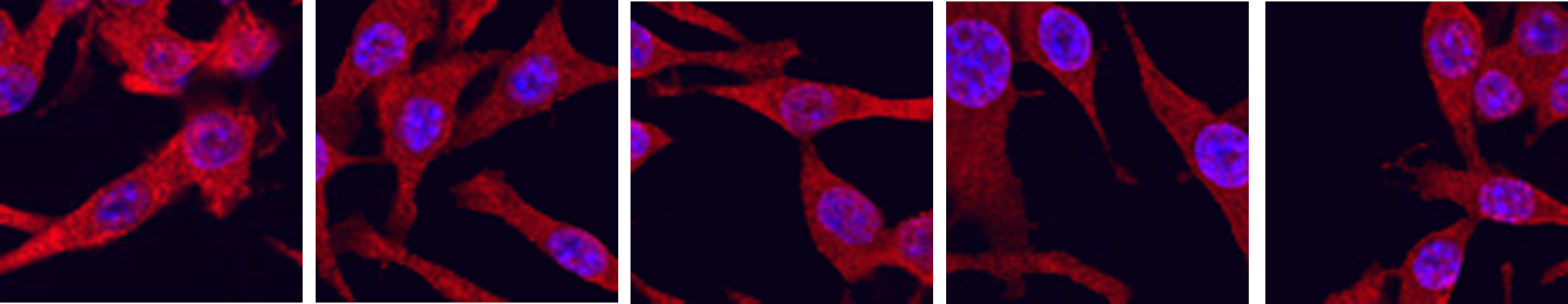


**PIC**

**QUIN**

**KMO**

**TDO-2**

**IDO-1**

**RAW cells: DAPI + FITC**


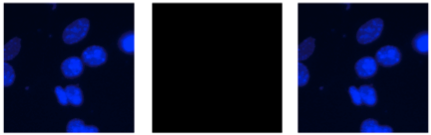

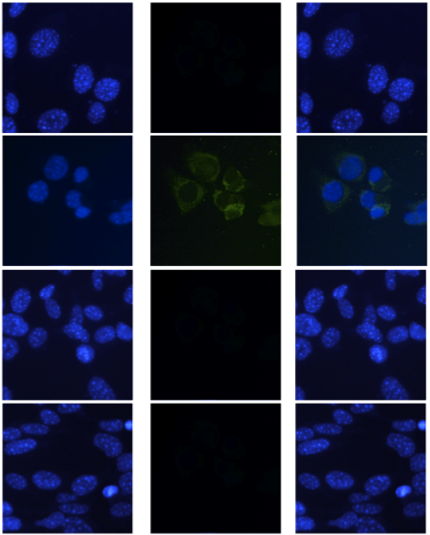

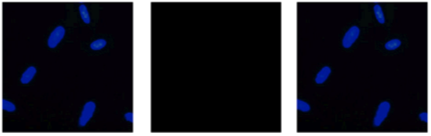

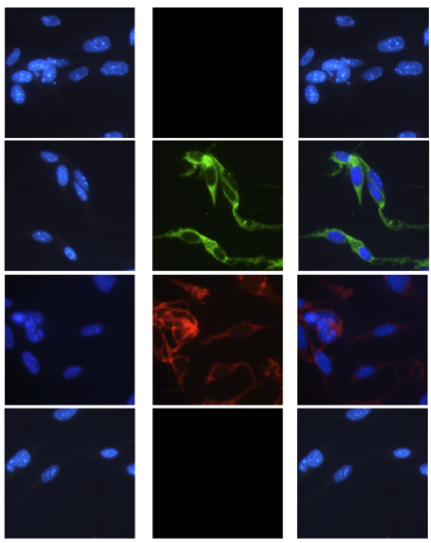


**IDO-1**

**TDO-2**

**KMO**

**QUIN**

**PIC**
